# No Stones, Some Groans, and Psychiatric Overtones with “Non-specific” Splenomegaly

**DOI:** 10.7759/cureus.4638

**Published:** 2019-05-10

**Authors:** Jonathan C Li, Emma Lundsmith

**Affiliations:** 1 Internal Medicine, Sidney Kimmel Medical College - Thomas Jefferson University, Philadelphia, USA; 2 Internal Medicine, Thomas Jefferson University Hospital, Philadelphia, USA

**Keywords:** splenic lymphoma, hypercalcemia of malignancy, hypercalcemia, splenomegaly, sonography

## Abstract

Hypercalcemia is a potentially life-threatening electrolyte imbalance that is commonly caused by hyperparathyroidism, supplement or medication use, and/or malignancy. Splenomegaly is commonly a non-specific finding, but in the setting of hypercalcemia, may provide diagnostic insight into the underlying pathology and warrant further evaluation. A 70-year-old man presented from his outpatient provider with serum calcium > 15 mg/dL with complaints of one-month fatigue, weakness, poor oral intake, 10 lbs. unintentional weight loss, and periodic confusion noted by his wife. He received an extensive inpatient workup which was non-diagnostic. Splenomegaly was observed on radiographic imaging and reported as “nonspecific”. Following discharge, denosumab was required to manage the hypercalcemia. Eventually, a diagnosis of primary splenic lymphoma was made months later. Laparoscopic splenectomy was planned but was advanced to an open laparotomy intraoperatively due to the rapid growth of the neoplasm. Early and close investigation of the spleen is warranted when splenomegaly presents in the setting of hypercalcemia and, as in this case, may prevent significant therapeutic burden.

## Introduction

Hypercalcemia is a potentially life-threatening electrolyte imbalance that is commonly caused by hyperparathyroidism, supplement or medication use, and/or malignancy [1-2]. Splenomegaly is commonly a non-specific finding, but in the setting of hypercalcemia, may provide diagnostic insight into the underlying pathology and warrant further evaluation.

## Case presentation

A 70-year-old Caucasian man presents from his outpatient provider with serum calcium > 15 mg/dL with complaints of one-month fatigue, weakness, poor oral intake, 10 lbs. unintentional weight loss, and periodic confusion noted by his wife. His past medical history is significant for hypertension, bronchial carcinoid tumor post-lobectomy 15 years prior, occupational asbestos exposure with calcified pleural plaques and low-risk prostate adenocarcinoma diagnosed 12 years prior, and recent negative biopsy one month prior to current admission. His medication is only amlodipine 10 mg daily and denies the use of any supplements. Family history is significant for lung cancer in his mother who was a heavy smoker and spinal cord astrocytoma in a daughter who is deceased. He denies any family history of sarcoidosis or autoimmune disease. Review of systems significant for right ankle soreness that began two weeks prior, and he denies fevers/chills, urinary symptoms, muscle aches, pain, fatigue, nausea/vomiting, or unintentional weight loss. Physical examination was non-contributory.

The patient’s admission labs were notable for normocytic anemia with hemoglobin of 13.2 g/dL (14.0-17.0 g/dL), hypercalcemia of 14.6 mg/dL (8.5-10.3 mg/dL) and acute kidney injury with a creatinine of 1.7 mg/dL from a baseline of 0.76 mg/dL. Electrocardiogram showed sinus rhythm without abnormal waveforms or intervals. With recommendations from nephrology and endocrinology, his hypercalcemia was managed with intravenous fluids, furosemide, calcitonin, and pamidronate. Diagnostic workup included a computed tomography (CT) of the abdomen and pelvis, skeletal survey, nuclear medicine bone scan, and serum protein electrophoresis /serum-free light chains analysis; all were normal with the exception of mild “nonspecific” splenomegaly to 14 cm and calcified mediastinal lymph nodes on CT. For bloodwork, his prostate-specific antigen was clinically stable at 5.9 (< 4.0 ng/mL), lactate dehydrogenase (LDH) 229 IU/L (125-240 IU/L), parathyroid hormone (PTH) 9 pg/mL (11-67 pg/mL), angiotensinogen-converting enzyme 35 U/L (8-52 U/L), aldolase 3.9 U/L ( ≤ 8.1 U/L), thyroid-stimulating hormone 2.0 uIU/mL (0.30-5.00 uIU/mL), 24-hour urine calcium of 1,027 mg/TVol ( ≤ 300 mg/TVol), PTH-related peptide (PTHrp) 17 pg/mL (14-27 pg/mL), and calcitriol (vitamin D, 1, 25 OH) of 133 pg/mL (18-72 pg/mL). Quantiferon gold was negative.

**Figure 1:**
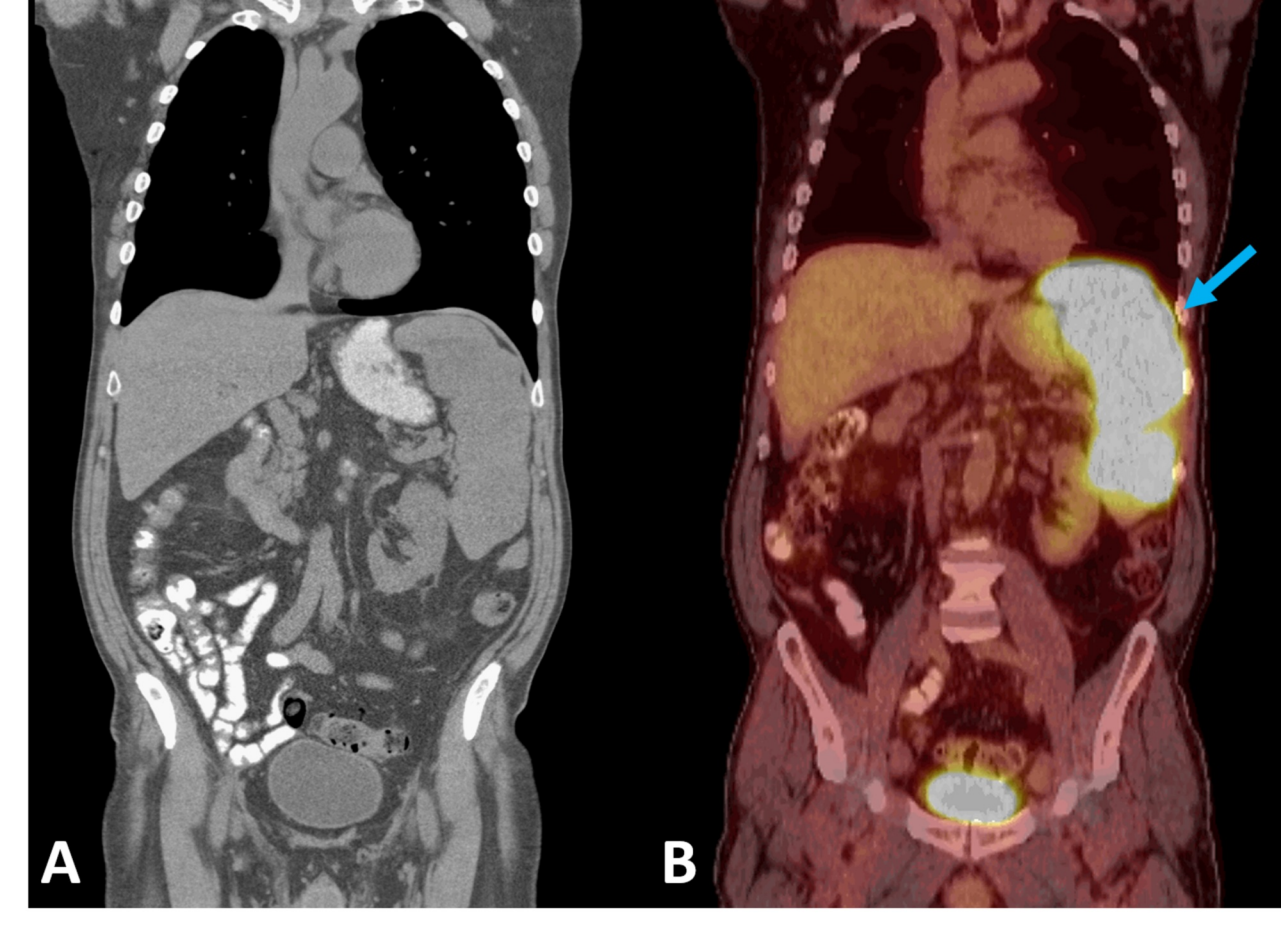
Left (A): CT of the chest, abdomen, pelvis without contrast read as: “No malignancy within the chest, abdomen, or pelvis, nonspecific splenomegaly, and calcified pleural plaques.” Right (B): Nuclear medicine PET tumor scan read as: “Splenomegaly with markedly hypermetabolic large foci of hypermetabolic activity, concerning for lymphoma (arrow). No additional or extra-nodal disease is seen in the chest, abdomen, and pelvis.” CT: computed tomography; PET: positron emission tomography

**Figure 2:**
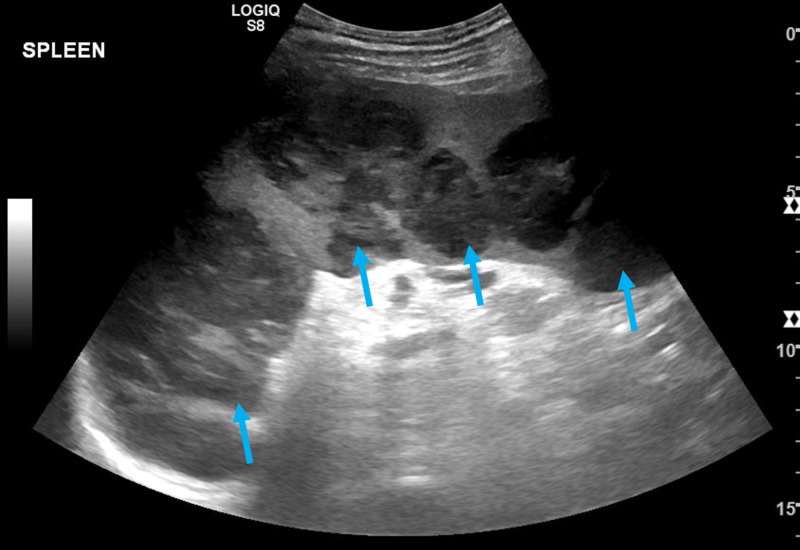
Abdominal ultrasound read as: “Splenic enlargement up to 18.5 cm in craniocaudal dimension. Multiple heterogeneous hypoechoic masses are noted throughout the spleen. The largest measures 9.6 x 7.8 x 8.9 cm. The masses demonstrate internal color Doppler flow.” Hypoechoic masses highlighted with arrows.

**Figure 3:**
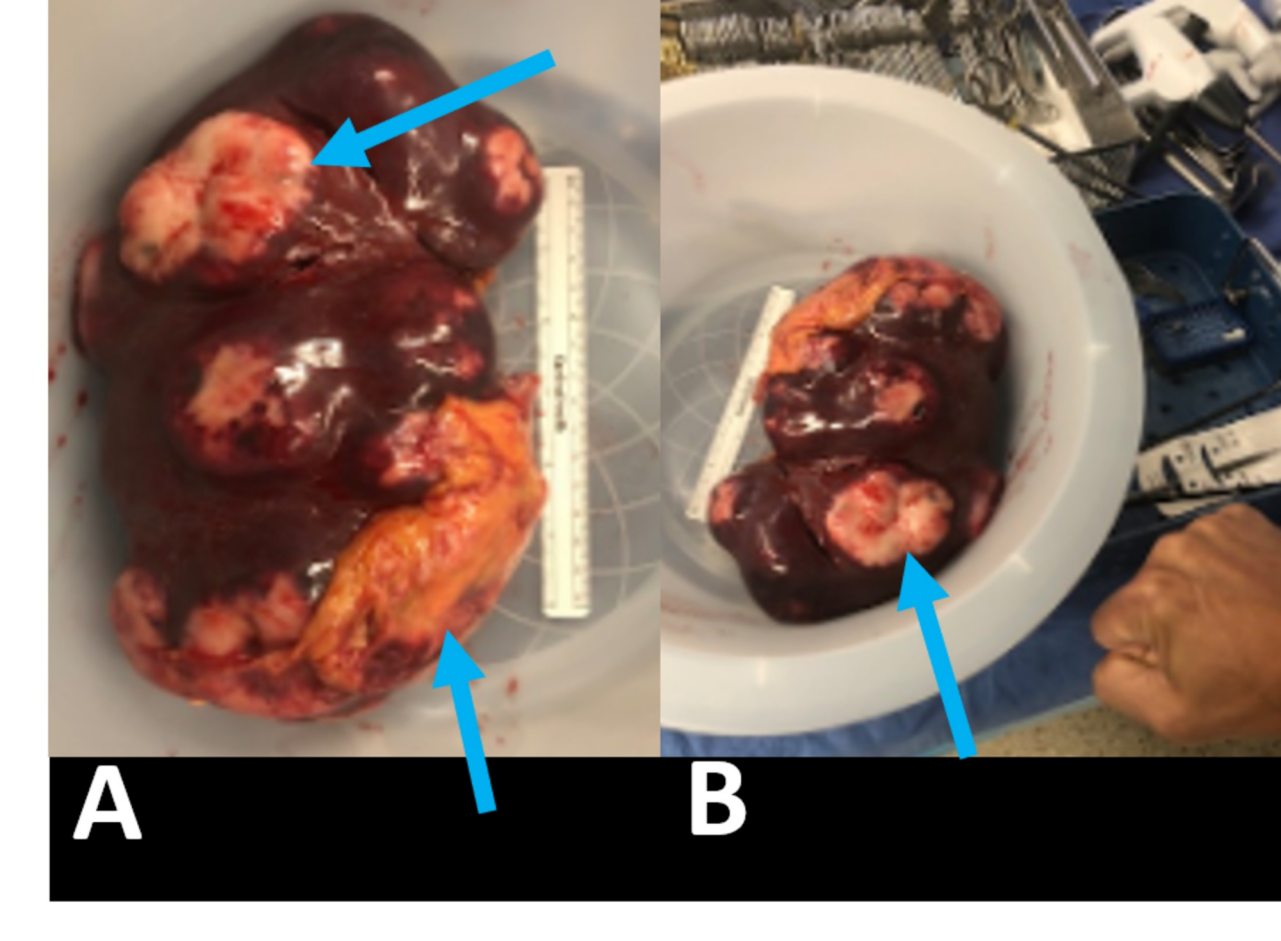
Post-splenectomy gross specimen with lymphomatous tissue (arrows). (A) Specimen compared to a 6-inch surgical ruler. (B) Specimen compared to a human fist.

**Figure 4:**
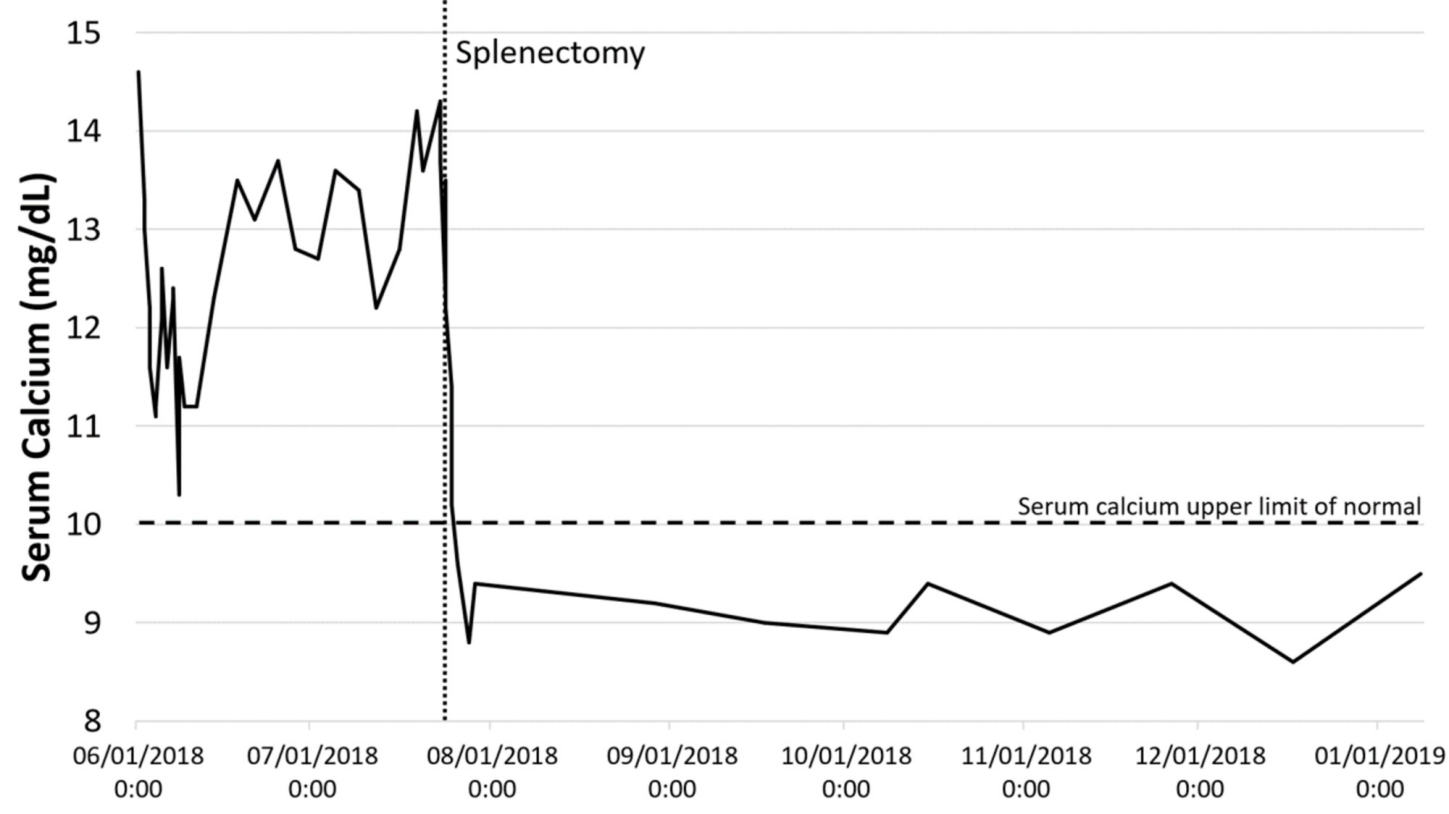
Serum calcium levels pre-splenectomy (6/1/2018 to 7/23/2018) and post-splenectomy (7/23/2018 To 1/31/2019).

The patient was discharged with a stable serum calcium of 10.3 mg/dL with outpatient follow-up from nephrology, endocrinology, and hematology/oncology. With high suspicion of hypercalcemia of malignancy, the patient was started on denosumab while continuing his workup. Peripheral blood flow cytometry, bone marrow biopsy, and fluorescence in situ hybridization analysis were unremarkable. One month after discharge, a positron emission tomography scan (PET-CT) was performed and was significant for splenomegaly with large foci of hypermetabolic activity, concerning for lymphoma (Figure 1). Twenty two days following PET-CT, a follow-up ultrasound was performed revealing splenomegaly to 18.5 cm and numerous hypoechoic masses throughout the spleen (Figure 2). The masses demonstrated internal color Doppler flow with the largest measuring 9.6 x 7.8 x 8.9 cm. Two months following presentation, a laparoscopic splenectomy was planned, however in the operating room, the spleen was found to have enlarged to approximately 22 cm in length with multiple internal tumors and omental adhesions requiring a conversion to an open laparotomy (Figure 3). Pathology revealed primary splenic large B-cell lymphoma (macronodular pattern). Large cells were positive for CD20, CD79a, and PAX-5; neoplastic cells were positive for BCL-2, and Ki-67 showed a high proliferation rate up to 90% among neoplastic cells. The patient’s hypercalcemia completely resolved following splenectomy, and he is currently receiving standard R-CHOP therapy (rituximab, cyclophosphamide, doxorubicin, vincristine, prednisone) for diffuse large B-cell lymphoma (DLBCL) (Figure 4).

## Discussion

DLBCL is the most common form of non-Hodgkins lymphoma, and 40% of systemic cases involve the spleen. However, primary splenic DLBCL (PS-DLBCL) is extremely rare with an estimated prevalence of < 1% [3]. Although PS-DLBCL is rare, it is the more common form of primary splenic lymphoma (PSL) [4]. Most cases of PS-DLBCL present with abdominal pain, elevated LDH, and splenic nodules [4]. An association between HCV and PS-DLBCL has been reported [4-5]. Our patient did not have a history of HCV, which was confirmed with serologic studies. 

Hypercalcemia can be stratified into mild (10-12 mg/dL), moderate (12-14 mg/dL), and severe (> 14 mg/dL). Severe hypercalcemia requires aggressive treatment with intravenous fluids (IVF), furosemide diuresis, calcitonin, and bisphosphonates [1]. Common causes include hyperparathyroidism, supplement or medication use, and/or malignancy [2]. As in this case, a low PTH supports low suspicion for primary hyperparathyroidism, although some tumors may secrete PTH [6]. An elevated 24-hour total urine calcium eliminated suspicion for hypocalciuric hypercalcemia. Additionally, our patient’s hypercalcemia was persistent and refractory to initial treatment, in the presence of an elevated calcitriol concern for hypercalcemia of malignancy was high. This diagnosis carries a mean survival rate of two to three months [1, 6]. Mechanisms for hypercalcemia of malignancy include bone resorption from metastasis, elevated vitamin D activation, or production PTHrp, all of which have been previously described as secondary to PSL [6-10]. A unique case of a PSL was also reported with completely normal labs (normal PTHrp, normal serum vitamin D) that resolved with splenectomy [11]. PSL associated with hypervitaminosis D and hypercalcemia is rarely reported [8-9, 12].

The most common causes for calcitriol-mediated hypercalcemia include granulomatous disease (most commonly sarcoidosis), hematologic malignancy, and mycobacterial infection [13]. Calcitriol mediates calcium homeostasis by promoting calcium absorption from the gut, reabsorption in the renal tubules, and resorption from bone [14]. Splenic involvement in sarcoidosis has a widely variable reported incidence [15-16]. Though commonly ordered, serum angiotensin-converting enzyme has poor sensitivity and specificity for sarcoidosis [17]. If doubts remain, imaging may be useful to differentiate splenic lymphoma from extrapulmonary, splenic sarcoid. Splenic sarcoidosis commonly shows homogeneous enlargement on ultrasound but may reveal small nodules with hypoechoic attenuation [16,18]. On contrast-enhanced CT, splenic sarcoidosis will also present as small hypodense nodules [16]. In comparison, splenic lymphoma rarely enhances with contrast on CT [18]. Although no guidelines exist on best imaging practices for working up splenomegaly; we recommend beginning with ultrasound to minimize radiation exposure and ease of use. Therefore, if assessment of splenomegaly in the setting of hypercalcemia reveals hypoechoic lesions on ultrasound, we recommend a follow-up study with contrast-enhanced CT. It is unclear why our patient developed PS-DLBCL. Occupational asbestos exposure is not associated with an increased risk for malignant lymphoma [19-20].

## Conclusions

Abnormal splenic morphology that is grossly nodular in appearance, as in our case, may warrant a more focused assessment for splenic pathology despite lacking classic hypoechoic lesions on radiographic imaging. Bedside ultrasound may yield an earlier diagnosis and minimize surgical morbidity in patients such as ours who required laparotomy rather than the originally planned laparoscopy. Contrast-enhanced CT is useful in differentiating splenic lymphoma from splenic sarcoidosis. PS-DLBCL is a rare entity primarily involving the spleen that may cause hypercalcemia. Systemic pathologies involving the spleen are not rare and splenomegaly as a clinical finding should not be overlooked.
